# Sweet Syndrome in an Elderly Man With Well-Controlled Human Immunodeficiency Virus

**DOI:** 10.7759/cureus.10330

**Published:** 2020-09-09

**Authors:** Steven M Mudroch, Craig Rohan, Nicholas G Conger, David A Lindholm

**Affiliations:** 1 Department of Medicine, Wright-Patterson Medical Center, Wright-Patterson Air Force Base, USA; 2 Department of Medicine, Wright State University, Dayton, USA; 3 Department of Dermatology, Wright-Patterson Medical Center, Wright-Patterson Air Force Base, USA; 4 Department of Medicine, Uniformed Services University of the Health Sciences, Bethesda, USA; 5 Department of Infectious Diseases, Wright-Patterson Medical Center, Wright-Patterson Air Force Base, USA

**Keywords:** sweet syndrome, hiv, acute febrile neutrophilic dermatosis

## Abstract

Acute febrile neutrophilic dermatosis, or Sweet syndrome, is a rare disorder associated with medications, underlying malignancy, or systemic inflammatory conditions. We present the case of a 71-year-old male living with well-controlled human immunodeficiency virus (HIV) on antiretroviral therapy, who presented with multiple painful, pseudo-vesicular, almost-necrotic appearing papules on his bilateral palms in the setting of constitutional symptoms and altered mental status. Biopsy of his palmar lesions revealed a dense, diffuse, dermal neutrophilic infiltrate consistent with Sweet syndrome. Infectious, rheumatologic, and oncologic work-up was negative. He was treated initially with intravenous immunoglobulin, prednisone, and dapsone; and he was continued on suppressive dapsone. He responded well clinically, but he relapsed multiple times in the setting of medication non-adherence before his ultimate diagnosis with sarcoidosis. A review of the literature of persons living with HIV and diagnosed with Sweet syndrome reveals no clear clinical association between the two despite plausible pathologic mechanisms. Patients living with HIV who are diagnosed with Sweet syndrome should be evaluated thoroughly for potential etiologies; the search for the underlying etiology of Sweet syndrome should go beyond their diagnosis of HIV.

## Introduction

Dermatologic conditions are common in persons living with human immunodeficiency virus (HIV). Although certain dermatologic conditions are more likely with a greater degree of immunosuppression, new-onset skin lesions can remain a diagnostic challenge even in well-controlled HIV [1]. We present the case of an elderly male with well-controlled HIV who developed acute febrile neutrophilic dermatosis (Sweet syndrome) and review the literature for case reports of patients with HIV and Sweet syndrome.

## Case presentation

**Figure 1:**
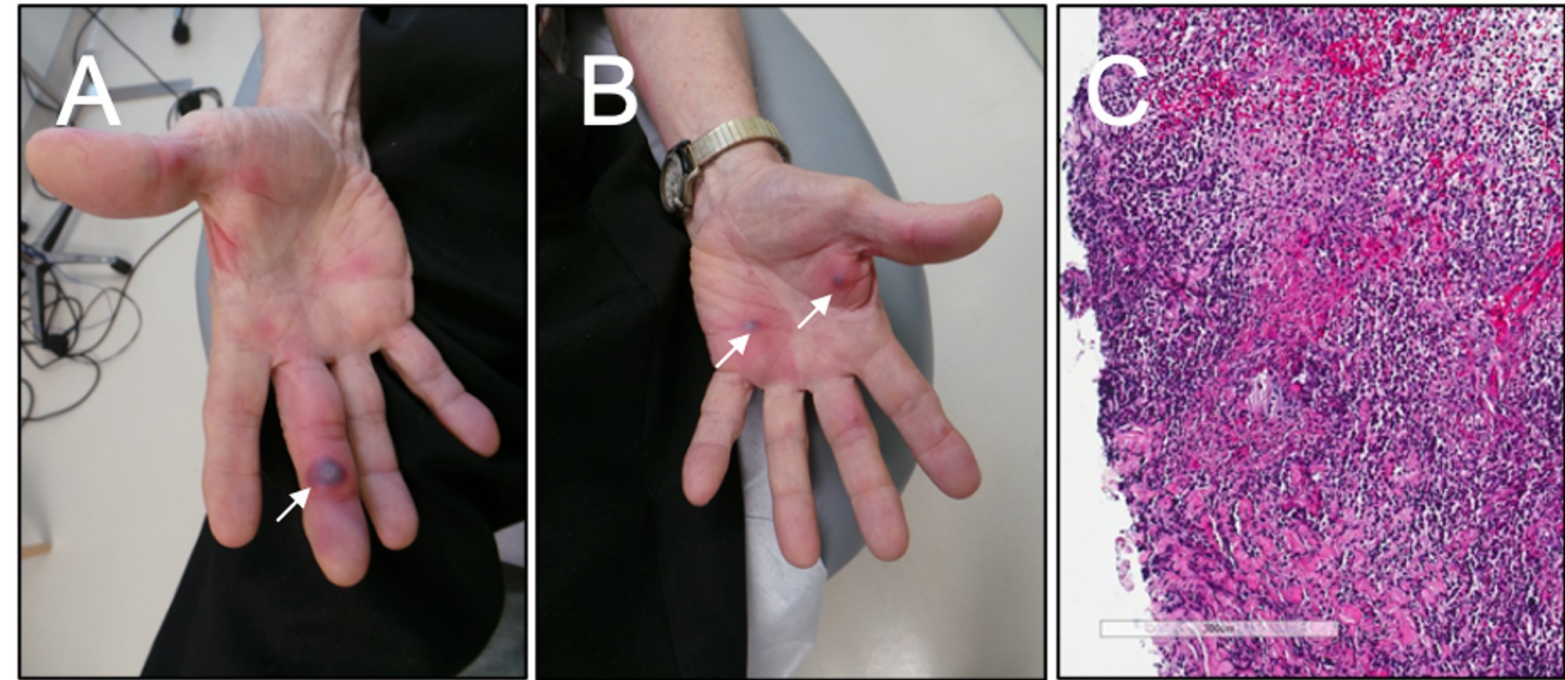
Initial clinical presentation and biopsy results. (A) Right and (B) left palms demonstrating scattered painful, pseudo-vesicular, almost-necrotic-appearing papules on his bilateral palms [arrows]. (C) Dense, diffuse, dermal neutrophilic infiltrate on biopsy from one of his palmar lesions. No features of vasculitis were identified.

A 71-year-old male with longstanding HIV, with a CD4 count of 320 cells/µL and viral load of 0 c/mL on tenofovir disoproxil fumarate, emtricitabine, and dolutegravir, presented to the Infectious Disease clinic with constitutional symptoms, altered mental status, and multiple painful, pseudo-vesicular, almost-necrotic-appearing papules on his bilateral palms (Figure 1). The lesions arose in the preceding few days to weeks and were associated with subjective fevers and night sweats. Recent medical history included completion of treatment for pulmonary histoplasmosis eight months prior, a transition from rilpivirine to dolutegravir as his antiretroviral anchor drug approximately one month prior, progressive confusion over several weeks, ongoing evaluation for failure to thrive, and treatment with azithromycin for an unknown infection five days prior at an outside facility. He was admitted to the hospital for further evaluation. Diagnostic work-up was notable for an elevated erythrocyte sedimentation rate (ESR) of 109 mm/hr. Evaluation for infectious etiologies included four sets of negative blood cultures and a negative rapid plasma reagin, hepatitis B virus DNA, hepatitis C virus antibody, Histoplasma urine antigen, and transthoracic echocardiogram. Cerebrospinal fluid (CSF) analysis revealed normal chemistries, no pleocytosis, and negative Venereal Disease Research Laboratory assay, cryptococcal antigen, and bacterial culture. Testing for *Neisseria gonorrhoeae* was not performed at the time of his evaluation. Rheumatologic evaluation included no evidence of C3 or C4 deficiency, cryoglobulins, antinuclear antibody, antineutrophil cytoplasmic antibody, or rheumatoid factor. Oncologic work-up included an unrevealing peripheral blood smear, serum paraneoplastic antibody panel, serum protein electrophoresis, and fecal occult blood testing. There were no masses on computed tomography of the head, chest, abdomen, or pelvis. Magnetic resonance imaging (MRI) of the abdomen revealed splenic enlargement without focal lesion and no evidence of underlying malignancy. MRI of the brain revealed no evidence of acute vasculitis.

**Figure 2:**
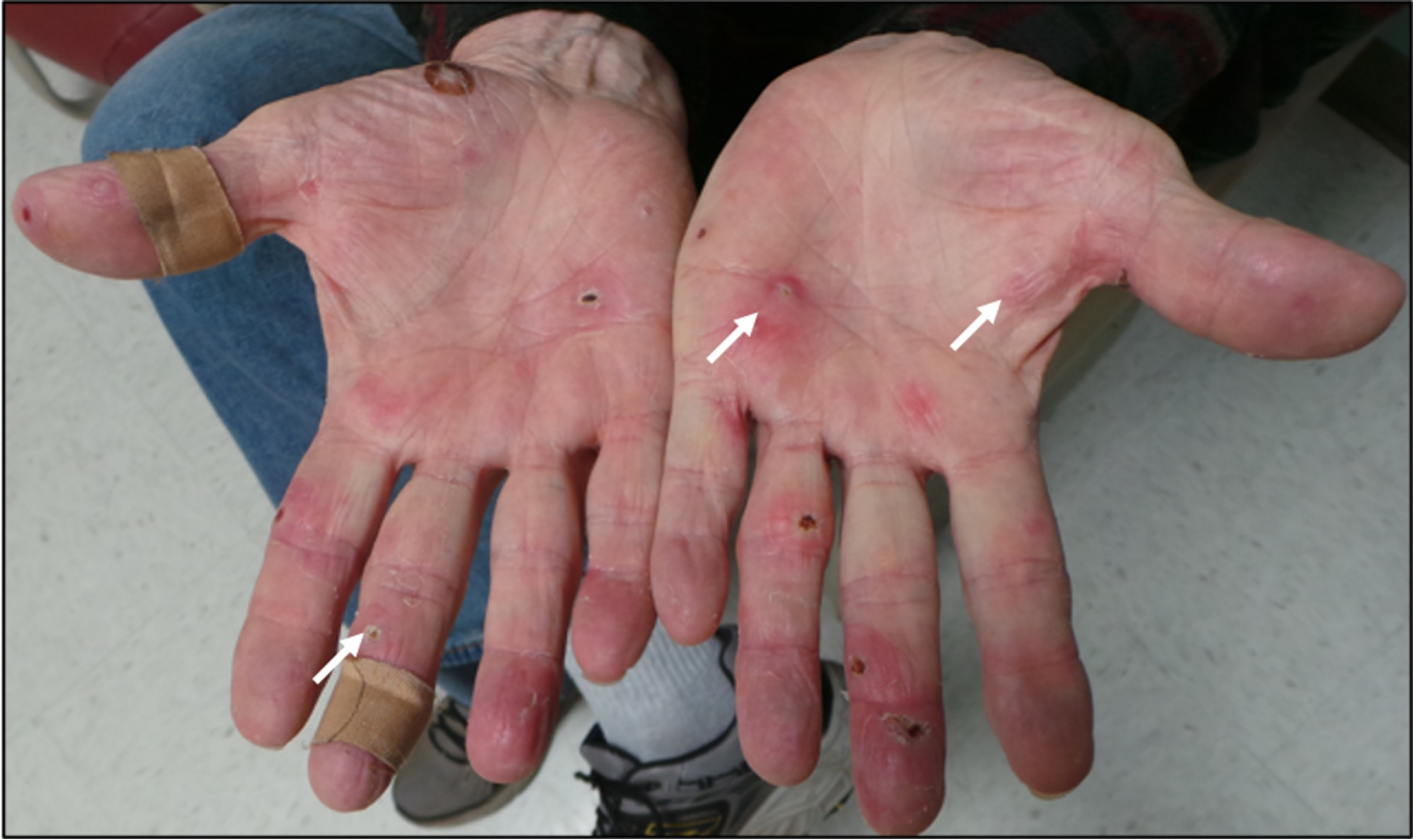
Follow-up clinical presentation. Interval improvement of initial palmar lesions [arrows] four weeks after initiation of treatment with IVIG, prednisone, and dapsone. IVIG: intravenous immunoglobulin.

A biopsy of the palmar lesions demonstrated a dense, diffuse, dermal neutrophilic infiltrate and papillary dermal edema (Figure 1). The biopsy was not consistent with vasculitis. The Gram stain was negative, and bacterial and fungal cultures exhibited no growth. Neuro-Sweet disease was considered given his altered mentation and cutaneous findings in the absence of another infectious, inflammatory, or malignant etiology. Initial treatment for his Sweet syndrome included intravenous immunoglobulin (IVIG), dapsone (started at 50 mg daily and titrated up to 100 mg daily), and prednisone (up to 60 mg daily). He demonstrated marked improvement in his skin lesions (Figure 2) and mental status by the time of hospital discharge eight days later. Prednisone was tapered off within two months, and his dapsone dose was tapered but continued as a suppressive, steroid-sparing therapy.

He did not have a recurrence of his cutaneous findings until a period of dapsone non-adherence four months after his initial diagnosis. Dapsone and prednisone were restarted and re-tapered. Over the following six months, he continued to have recurrent flares of Sweet syndrome, thought to be secondary to medication non-adherence. Throughout this time, his viral load remained suppressed, and his CD4 count remained stable on an integrase-inhibitor-based regimen. 

Our patient was unable to be weaned off suppressive dapsone, prompting consideration for an unresolved, underlying etiology. Newly enlarged inguinal lymph nodes were found on palpation one year after the diagnosis of Sweet syndrome. Positron emission tomography (PET) demonstrated mild 18F-fluorodeoxyglucose (FDG) avidity in his enlarged spleen, inguinal and retroperitoneal lymph nodes, and skeleton, but an excisional lymph node biopsy did not find evidence of lymphoma. Colonoscopy was also negative.

With the recurrence of bilateral palmar lesions almost two years after the initial presentation, a repeat punch biopsy was performed, revealing non-necrotizing granulomatous inflammation and leading to a diagnosis of sarcoidosis. He ultimately died 2.5 years after his initial presentation, with an unknown cause of death. 

## Discussion

Sweet syndrome is defined by major and minor criteria. The major criteria include acute, tender, erythematous, or violaceous plaques or nodules as well as neutrophilic dermal infiltrate in the absence of leukocytoclastic vasculitis. The minor criteria include antecedent fever or infection; concomitant fever, arthralgia, conjunctivitis, or malignancy; leukocytosis; good clinical response to systemic corticosteroids rather than antibiotics; and increased ESR [2]. Our patient met the case definition of Sweet syndrome based on both major criteria and three minor criteria (antecedent fever, good clinical response to steroids, and increased ESR). Classically, Sweet syndrome is more common in women in the fourth to sixth decades of life [1]; our patient is the oldest of the reported cases of Sweet syndrome in an individual living with HIV. 

The Sweet syndrome has been described aptly as a “diagnosis searching for a cause,” as the diagnosis should prompt evaluation for an underlying etiology [1]. Potential etiologies include infections, malignancy, autoimmune/inflammatory conditions, medications, and pregnancy; Sweet syndrome has also been described as classic or idiopathic if it is neither malignancy-associated nor drug-induced [1,2]. 

We performed a literature search using PubMed with search terms “HIV AND Sweet” or “HIV AND Acute febrile neutrophilic dermatosis,” and identified 17 other published cases from 1994 to 2020 of Sweet syndrome in patients with HIV infection (Table 1). One of these cases is mentioned in the context of a larger study on histopathological findings, but no further supporting details were available for tabulation [3]. 

**Table 1:** Cases of Sweet syndrome in persons living with HIV, arranged by the year of publication 3TC: lamivudine, ABC: abacavir, ART: antiretroviral therapy, ATV: atazanavir, AZT: zidovudine, BID: twice daily, D4T: stavudine, DDC: zalcitabine, DTG: dolutegravir, EFV: efavirenz, F: female, FTC: emtricitabine, HIV: human immunodeficiency virus, IRIS: immune reconstitution inflammatory syndrome, IVIG: intravenous immunoglobulin, LPV: lopinavir, M: male, NFV: nelfinavir, NR: not reported, NSAID: non-steroidal anti-inflammatory drug, RTV: ritonavir, SQV: saquinavir, TID: three times daily, TDF: tenofovir, UD: undetectable, VL: viral load.

Author [ref]	Year	Age (year)	Sex	CD4 (cells/ µL)	VL (c/mL)	Etiology	ART at Sweet onset	Treatment
Hilliquin et al. [4]	1992	30	F	283	NR	HIV	None	Prednisone 30 mg/day; colchicine 1 mg/day
Berger et al. [5]	1994	43	M	<50	NR	G-CSF, Kaposi sarcoma	None	Prednisone 40 mg/day; potassium iodide solution; hydroxychloroquine 200 mg BID
Berger et al. [5]	1994	54	M	143	NR	Unknown	None	Potassium iodide solution; stop NSAID and glyburide
Bevilacqua et al. [6]	1999	41	F	187	550,000	IRIS (possible), Pneumocystis pneumonia	AZT, DDC, SQV	Prednisone 1 mg/kg
Brady et al. [7]	1999	3 months	M	368	NR	HIV (new diagnosis)	None	Polysporin ointment, topical; viscous lidocaine, topical
Del Giudice et al. [8]	2004	47	F	173	140,000	ABC	3TC, NFV, ABC	Stop ABC
Tan et al. [9]	2006	46	M	414	NR	Influenza vaccination	None	Prednisolone 1 mg/kg/day
Inamadar and Anitha [10]	2008	30	F	450	NR	HIV	None	Dapsone 100 mg/day; prednisone 1 mg/kg/day
Cabanillas et al. [11]	2008	35	M	285	100,000	HIV (new diagnosis)	None	Prednisone 50 mg/day
Johnson et al. [12]	2008	37	M	530	UD	Syphilis	FTC, TDF, ATV, RTV	Corticosteroids; treat syphilis
Haddow and Lehloenya [13]	2011	36	F	217	21,000	IRIS	D4T, 3TC, LPV, RTV	NR
Haddow and Lehloenya [13]	2011	51	F	126	5,000	IRIS	D4T, 3TC, EFV	Topical steroids; indomethacin
Deasy et al. [14]	2012	44	M	459	96,200	Bone marrow dyscrasia	ABC, 3TC, EFV	Colchicine 500 mcg TID (dapsone and prednisone ineffective)
Corral et al. [15]	2014	38	F	620	28,000	HIV	3TC, EFV	Betamethasone
Rajendran et al. [16]	2014	67	F	163	NR	Unknown	None	Prednisone 1 mg/kg/day
Dong et al. [17]	2020	47	F	70	NR	IRIS	3TC, TDF, DTG	Thalidomide 100 mg/day
Current case	2020	71	M	320	UD	Unknown	TDF, FTC, DTG	Prednisone 60 mg/day; IVIG; dapsone 100 mg/day

A review of the published cases highlights important unique proposed etiologies for Sweet syndrome in those living with HIV: immune reconstitution inflammatory syndrome (IRIS) in 25% of cases [6,13,17], AIDS-defining conditions (i.e., Kaposi sarcoma and pneumocystis pneumonia) in two cases [5,6], and abacavir therapy in one case [8]. Other notable attributions include syphilis [12], influenza vaccination [9], bone marrow dyscrasia [14], and treatment with G-CSF [5]. No cause was determined in two of the cases.

The published cases do not indicate a clear association between Sweet syndrome and CD4 cell count (range: <50 to 530 cells/µL), the degree of viral suppression (range: undetectable to 550,000 c/mL), or HIV itself. At least two reported cases have identified Sweet syndrome as a presenting manifestation of HIV infection [7,11], and others postulate an association with HIV after other etiologies have been ruled out [4,10,15]. No definitive pathogenic mechanism has been elucidated to connect the two, though some hypothesize that HIV creates an environment more favorable to the pathogenesis of Sweet syndrome [6] through a hypersensitivity response with neutrophil chemotaxis [7,13], altered Type 1 and Type 2 helper T-cell response [14,17], and the presence of HIV trans-activating proteins [11]. Despite one-third of reported cases directly attributing HIV itself as the etiology of Sweet syndrome, larger studies involving nearly 1700 persons living with HIV and reporting to dermatology clinics have not identified Sweet syndrome as a presenting skin disorder [18,19].

The underlying etiology of our patient’s Sweet syndrome remains unknown. His recent transition to a dolutegravir-based regimen is notable given that one other case also reported initiation of a dolutegravir-based regimen [17], though that case was attributed ultimately to IRIS, and our patient had longstanding control of HIV. Sarcoidosis may have contributed, as it has been identified previously as a possible associated condition [20]. Although he had evidence of a systemic inflammatory condition at the time of his original presentation, the contribution of sarcoidosis is questionable, as there was no evidence of granuloma formation on biopsy at that time.

There was significant heterogeneity in treatment approaches for Sweet syndrome in those living with HIV, though a majority of treatments involved systemic anti-inflammatory and/or immunosuppressive therapies [4-7,9-11,13-16,20]. Duration of treatment was also variable, but successful treatments ranged from as little as ten days with a course of saturated potassium iodide solution [7], to two months of prednisone [4], often with a complete response to treatment [2].

## Conclusions

Sweet syndrome is classically associated with medications, malignancy, or systemic inflammatory conditions. It is unclear whether HIV is a true precipitating factor. Review of the literature has noted attributions of Sweet syndrome to IRIS, AIDS-defining conditions, medications, vaccinations, other infections, and altered bone marrow function. Our report demonstrates that Sweet syndrome should be part of the differential diagnosis in persons living with HIV who present with new skin plaques or nodules and constitutional symptoms, regardless of the patient’s age and degree of HIV control. The search for the underlying etiology of Sweet syndrome in patients living with HIV should go beyond their diagnosis of HIV.
